# Retraction: Efficacy and Safety of Peptide Receptor Radionuclide Therapy for the Treatment of Pancreatic Neuroendocrine Tumors: A Systematic Review and Meta-Analysis of Comparative Studies

**DOI:** 10.7759/cureus.r178

**Published:** 2025-04-04

**Authors:** Jun Zhao, Xiaxia Pei, Yumin Li

**Affiliations:** 1 Department of General Surgery, Second Hospital of Lanzhou University, Lanzhou, CHN; 2 Department of Oncology, Second Hospital of Lanzhou University, Lanzhou, CHN; 3 Key Laboratory of the Digestive System Tumors of Gansu Province, Second Hospital of Lanzhou University, Lanzhou, CHN

This article has been retracted by the Editors-in-Chief. The authors requested retraction due to significant concerns regarding the quality of evidence included in the review. Specifically, several of the studies analyzed were abstract-only publications that had not undergone peer review, thereby undermining the validity and reliability of the findings. The Editors-in-Chief agree with these concerns, therefore the article has been retracted.

